# Global Transcriptome Profiling Identified Transcription Factors, Biological Process, and Associated Pathways for Pre-Harvest Aflatoxin Contamination in Groundnut

**DOI:** 10.3390/jof7060413

**Published:** 2021-05-26

**Authors:** Pooja Soni, Arun K. Pandey, Spurthi N. Nayak, Manish K. Pandey, Priya Tolani, Sarita Pandey, Hari K. Sudini, Prasad Bajaj, Jake C. Fountain, Prashant Singam, Baozhu Guo, Rajeev K. Varshney

**Affiliations:** 1 Center of Excellence in Genomics & Systems Biology (CEGSB), International Crops Research Institute for the Semi-Arid Tropics (ICRISAT), Hyderabad 502324, India; poojasoni.sps@gmail.com (P.S.); m.pandey@cgiar.org (M.K.P.); priyatolani90@gmail.com (P.T.); isarita06@gmail.com (S.P.); p.bajaj@cgiar.org (P.B.); 2 Department of Genetics, Osmania University, Hyderabad 500007, India; prashantsingam@gmail.com; 3 College of Life Science, China Jiliang University (CJLU), Hangzhou 310018, China; pandeyarun@cjlu.edu.cn; 4 Department of Biotechnology, University of Agricultural Sciences, Dharwad 580005, India; nayaksn@uasd.in; 5 Theme-Integrated Crop Improvement, Research Program-Asia, International Crops Research Institute for the Semi-Arid Tropics (ICRISAT), Hyderabad 502324, India; h.sudini@cgiar.org; 6 Department of Biochemistry, Molecular Biology, Entomology, and Plant Pathology, Mississippi State University, Starkville, MS 39762, USA; jcf416@msstate.edu; 7 Crop Genetics and Breeding Research Unit, USDA-ARS, Tifton, GA 31793, USA; baozhu.guo@usda.gov; 8 State Agricultural Biotechnology Centre, Centre for Crop and Food Innovation, Food Futures Institute, Murdoch University, Murdoch, WA 6150, Australia

**Keywords:** aflatoxin, *Aspergillus flavus*, biotechnology, food safety, gene expression, genomics, pre-harvest aflatoxin contamination (PAC), RNA-seq, transcriptome analysis

## Abstract

Pre-harvest aflatoxin contamination (PAC) in groundnut is a serious quality concern globally, and drought stress before harvest further exacerbate its intensity, leading to the deterioration of produce quality. Understanding the host–pathogen interaction and identifying the candidate genes responsible for resistance to PAC will provide insights into the defense mechanism of the groundnut. In this context, about 971.63 million reads have been generated from 16 RNA samples under controlled and *Aspergillus flavus* infected conditions, from one susceptible and seven resistant genotypes. The RNA-seq analysis identified 45,336 genome-wide transcripts under control and infected conditions. This study identified 57 transcription factor (TF) families with major contributions from 6570 genes coding for bHLH (719), MYB-related (479), NAC (437), FAR1 family protein (320), and a few other families. In the host (groundnut), defense-related genes such as senescence-associated proteins, resveratrol synthase, seed linoleate, pathogenesis-related proteins, peroxidases, glutathione-S-transferases, chalcone synthase, ABA-responsive gene, and chitinases were found to be differentially expressed among resistant genotypes as compared to susceptible genotypes. This study also indicated the vital role of ABA-responsive *ABR17*, which co-regulates the genes of ABA responsive elements during drought stress, while providing resistance against *A. flavus* infection. It belongs to the PR-10 class and is also present in several plant–pathogen interactions.

## 1. Introduction

Aflatoxins are strong cancer-causing and teratogenic mycotoxins produced in crops such as groundnut, maize, cottonseed, chilies, and tree nuts upon infection by *Aspergillus flavus* and *A. parasiticus*. Groundnut or peanut (*Arachis hypogaea* L.) is the most susceptible crop to aflatoxin contamination under favorable conditions [1,2]. Aflatoxin exposure can have significant negative impacts on human health. It is also considered as a growth development retardant in the young [3] and an immune suppressor [4], contributing to susceptibility to AIDS [5]. In groundnut, Aflatoxin B_1_ (AFB_1_) produced by the *A. flavus* and *A. parasiticus* is believed to be the essential driver of liver disease, alongside ongoing hepatitis B and C infections [6] and intense aflatoxicosis [7,8]. The resistance mechanisms to aflatoxin contamination in groundnut can be delineated into resistance to pre-harvest aflatoxin contamination (PAC), post-harvest aflatoxin contamination, and fungal colonization on seeds [2,9,10]. The PAC is a vital segment of aflatoxin which, if ignored, increases in intensity during post-harvest operations such as packing, transportation and storage [11,12,13]. In this condition, plants look healthy from the outside but the contamination can be seen inside the pods, is possible only after harvest. The inoculum carried in the pre-harvest conditions are the major source of contamination found at post-harvest and storage stages. Abiotic stresses, especially drought and heat, exacerbate pre-harvest aflatoxin production in groundnut [14,15]. Pre-harvest aflatoxin contamination may be diminished in groundnut by identifying drought resistant cultivars, adequate water system facilities, and best post-management practices [15,16,17]. Under drought conditions, the cracking of pod walls may result in increased penetration of *A. flavus* and subsequent aflatoxin contamination in the kernels [18]. The drought conditions also decreased the production of phytoalexin, due to low kernel water activity, which plays an important role in defense mechanisms against aflatoxin contamination [19]. Thus, drought is a factor which plays a key role in exacerbating aflatoxin contamination in groundnut [20,21].

The next-generation sequencing (NGS) based transcriptomic approach examines resistance mechanisms by identifying novel genes and pathways [22]. Few reports are available related to expression profiles for selected tissues of plant development and improvement in groundnut utilizing microarray innovation [23,24] and transcriptome sequencing [25,26]. One of the previous transcriptome analysis studies highlighted the key role of fatty acid and abscisic acid (ABA) biosynthesis pathways, in addition to identifying ABR1, a repressor and a susceptibility factor which acts on ABA signaling pathways and promote PAC [21]. However, the unavailability of reference genomes either restricted such studies or could not provide conclusive information during in-vitro investigation of *Aspergillus* disease with different degrees of aflatoxin contamination in groundnut [27]. Nevertheless, reference genomes have now become available not only for diploid progenitors [28,29] but also for both of the subspecies of cultivated groundnut [30,31,32]. These genome assemblies are great sources for understanding the structural and functional behavior of complex traits including aflatoxin contamination [33,34,35]. In view of the above, the current RNA-seq study was conducted for developing a better understanding of the molecular mechanism in groundnut for PAC resistance against *A. flavus* using seven resistant genotypes and one susceptible genotype under infected and control conditions in the glasshouse conditions. This study provides putative candidate genes that were differentially expressed in these cultivars, and metabolic pathways associated with resistance to fungal infection and PAC. The potential candidate genes can be targetted for further validation and for developing marker assays to accelerate breeding for low aflatoxin contamination in groundnut.

## 2. Material and Methods

### 2.1. Plant and Fungal Material

Seven resistant genotypes (ICGV 91284, ICGV 94379, ICGV 91324, ICGV 91278, ICGV 91315, ICGV 93305, and J 11) and one susceptible genotype (JL 24) were used in this transcriptome study for understanding PAC resistance mechanisms. These seven resistant genotypes were safe, within the permissible level (<15 ppb) of aflatoxin four to eight weeks after harvest. Fungal cultures of highly toxigenic strains of *A. flavus* (isolate of 11-4) were used for the inoculation. Characterization of the toxigenic *A. flavus strain* (AF 11-4) was done at the Groundnut Pathology Unit, ICRISAT. The AF 11-4 strain was maintained and sub-cultured on Potato Dextrose Agar (PDA) plates, and was further used in this present study for field inoculation. A conidial suspension was prepared and adjusted to a concentration of 1 × 10^6^ spores/mL, after 7 days of incubation at 25 °C.

### 2.2. Experimental Setup

The seeds of all the eight genotypes were disinfected with 0.1% mercuric chloride solution for 2–5 min with gentle shaking and 1 min in 70% ethanol followed by rinsing with sterile water three to four times. Care was taken to completely sterilize the soil mixture (red soil + sand (2:1)) in a horizontal autoclave. The sterilized soil mixture was placed in pots of 10” diameter with 5–7 g of diammonium phosphate (DAP) in each pot. Before sowing, the seeds were treated with systemic fungicide SAAF at 1 g/kg of seeds. In a glasshouse, 10 plants for each genotype were maintained for control and treatment. Both the control and infected sets were allowed to grow normally for 30 days. The treatment set was inoculated with the *A. flavus* inoculum that was grown on the autoclaved sorghum grains 30 days after sowing (DAS) at the pod zone. The second inoculation was made 45 DAS followed by a third inoculation at 60 DAS. Two irrigations per week were given until 75 DAS, and a dry spell was mimicked from 75 DAS to 90 DAS. The plants were irrigated optimally to prevent soil moisture stress during all of the crop growth stages except for imposing the end-of-season (3–4 weeks before harvest) drought, which favored *A. flavus* penetration and subsequent entry into pods. Water was applied to the pots by hand using a hose. Each time approximately 10% more water was applied than the pot could hold, ensuring that the applied water percolated into the soil rather than overflowing from the top of the pot. A life-saving irrigation was given 90 DAS and harvesting was carried out at 120 DAS. Further harvested seeds samples were used for toxin estimation and RNA-seq analysis.

### 2.3. Aflatoxin Quantification in Control and Infected Samples

Quantitative estimation was done for total aflatoxins under control and infected conditions following the protocol of Waliyar et al. [36]. An indirect competitive enzyme-linked immune sorbent assay (ELISA) approach was used, and polyclonal antibodies were produced against AFB_1_) [36]. To do so, we utilized polyclonal antibodies against AFB_1_ for the quantitative assessment of all of the aflatoxins. For polyclonal antibody production, aflatoxin B_1_-bovine serum albumin (AFB_1_-BSA) was acquired from Sigma-Aldrich (Catalog No. 6655, Suffolk, NY, USA).

### 2.4. RNA Extraction, Illumina Sequencing and Data Pre-Processing

For RNA isolation, frozen seeds were homogenized into a fine powder using a chilled mortar and pestle. Total RNA was extracted from 5 g of seeds using the “NucleoSpin^®^ RNA Plant” kit (Macherey-Nagel, Düren, Germany) according to the manufacturer’s instructions. A quality check of RNA samples was performed using a Nanodrop 1000 spectrophotometer (Thermo Fisher Scientific Inc, Waltham, MA, USA) and an Aglient RNA 6000 Nano chip on an Agilent 2100 Bioanalyzer (Agilent technologies, Palo Alto, CA, USA). The RNA samples with a 260/280 ratio of 1.8 to 2.1 or a 260/230 ratio of 2.0 to 2.3, and a RIN (RNA integrity number) value of >8.0, were used for cDNA library preparation. A total of 16 libraries (8 genotypes × 2 treatment levels) were sequenced on the NextSeq 500 platform to generate 75-base paired-end reads at Genotypic Technology Pvt. Ltd. (Bengaluru, India). 5 μg of the total RNA pooled in an equivalent amount from two replicates were utilized for the development of a cDNA library utilizing an mRNA-Seq test prep unit (Illumina Inc., San Diego, CA, USA). The raw reads were subjected to quality filtering using NGSQCbox [37] and Trimmomatic v0.33 [38] to remove low-quality sequencing reads with ambiguous nucleotides and any adapter contamination. The read quality was assessed using FastQC.

### 2.5. Trimming, Alignment and Functional Annotation of Sequences

Sequencing data were processed post-trimming following approaches mentioned in Clevenger et al. [39] and Chen et al. [29]. Tophat2 v2.1.1 was used for alignment against two diploid progenitor genomes of cultivated groundnut with parameters set to default [40]. The aligned reads were then separated for reads aligned on *A. duranensis* (A genome) and *A. ipaensis* (B genome) and assembled separately based on the genome-guided approach using trinity v2.2.0 [41]. Further, the unaligned reads were also assembled using the de novo approach. The assembled transcripts for each sample were then filtered for redundancy using the evidential gene pipeline [42]. The filtered reads of the 16 resultant samples were mapped onto assembled transcripts and the expression level of transcripts was estimated in terms of fragments per kilobase of transcript per Million (FPKM) using the Cuffdiff program within Cufflinks v2.2.1 [43]. A transcript was considered to be expressed when FPKM ≥ 1 in at least one sample. For a transcript to be significant, |log_2_ (fold change)| ≥ 2 and *p*-values ≤ 0.05 were considered. In addition, to visualize differential gene expression patterns between the 8 control and 8 infected genotypes, a Venn diagram was constructed.

### 2.6. Transcript Annotations, Pathway Assignment and Annotations of Transcription Factors

Alignment was performed to identify significant hits for genes exposed to the BLASTX similarity search against NCBI non-redundant (nr) protein database taxon Viridiplantae with a cut-off of E-value ≤ 1 × 10^−5^. BlastX results were then used to distinguish gene ontology (GO) annotation and pathways through Blast2GO v5 [44]. By using a tissue specificity index (τ) as described by Yanai et al. [45], the sample-specific genes were identified with following the formula:

$$\tau =\frac{\sum_{i=1}^{N}\left(1-x_{i}\right)}{N-1}$$

where N is the number of samples and $x_{i}$ is the expression value of a gene normalized by most extreme value across all samples. The estimation of τ goes from 0 to 1, where the higher the value more plausible the specific expression at that stage. Transcription factors (TFs) are key proteins that control the regulation of the gene expression in various phases of the life cycle. The TFs were annotated in transcriptome sequencing data generated on eight infected and control genotypes using BLASTX with the cut off E-value ≤ 1 × 10^−10^ by comparing plant transcription factors (PlantTFDB) [46].

## 3. Results

### 3.1. Transcriptome Sequencing and Gene Expression Analysis

**Figure 1:**
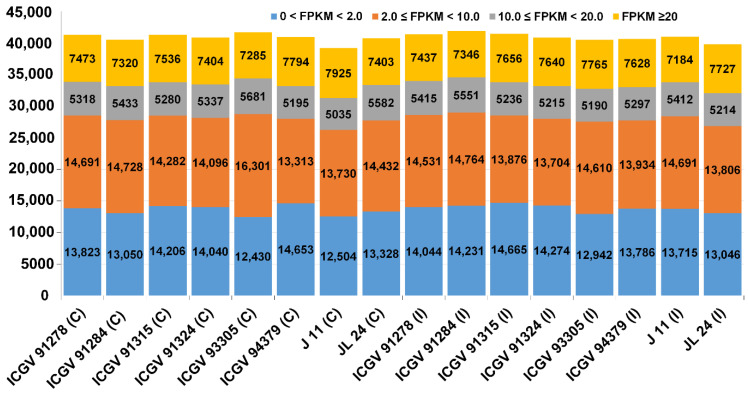
Distribution of genes expressed in 16 samples. Based on expression level, genes were grouped into four classes (FPKM < 2, 2 ≤ FPKM < 10, 10 ≤ FPKM < 20 and FPKM > 20) in sixteen samples.

A total of 971.63 million paired-end reads were produced from 16 samples (Table 1) and 941.93 million paired-end reads (96.94% of the total paired-end reads) were retained for the downstream process after the filtration of low-quality reads. An average of 94.13% of the filtered reads were mapped to the assembled transcripts. Of the 56,239 transcripts identified among various tissue samples, 45,336 genes (Table S1) had an abundance of FPKM ≥ 1 in at least one sample. The details of annotation, gene ontology (GO) IDs and names, and the corresponding putative pathways of each of 45,336 transcripts are provided in Table S1. Further, based on expression levels, they were ordered into four significant classes (FPKM < 2, 2 ≤ FPKM < 10, 10 ≤ FPKM < 20 and FPKM > 20) in sixteen samples. The distribution of genes expressed in 16 samples under these categorizations based on their expression levels (low, moderate, and high) has been shown in Figure 1.

Upon examining the genes with FPKM ≥ 20, the highest number of highly expressed genes were identified in the moderately resistant genotype J 11 (C) (7925 genes) and susceptible genotype ICGV 93305 (I) (7765 genes). Of the total 56,239 genes identified across genotypes, the highest number (41,697) of genes was expressed in ICGV 93305 (C) followed by 41,892 genes in ICGV 91284 (I) and 41,433 genes in ICGV 91315 (I) (Figure 1). Further, 426 stably expressed genes were identified across tissues and samples based on coefficient of variation (CV). In light of the large diversity of analyzed tissues in this study, the top constitutively expressed genes which displayed a CV between 2.50 to 3.45% were marked for further investigation into their pre-harvest aflatoxin resistance. These genes belong to ABA-responsive, pathogenesis-related proteins (PR proteins), peroxidases, glutathione-S-transferases, seed linoleate, chalcone synthase, defense-related genes, and chitinases categories.

### 3.2. Clustering and Principal Component Analysis

**Figure 2:**
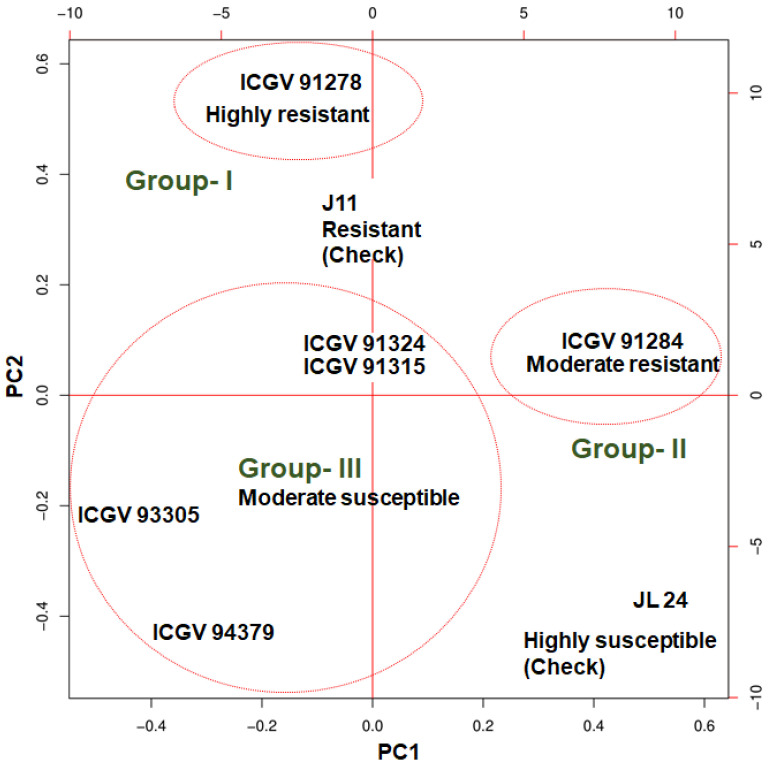
Principal component analysis depicting correlation among samples based on gene expression data. Group 1 (ICGV 91278), highly resistant, group 2 (ICGV 91284) moderately resistant, group 3 (ICGV 91324, ICGV 91315, ICGV 93305, ICGV 94379), moderately susceptible, in comparison to highly susceptible check JL24.

Principal component analysis (PCA) was performed to explore the relationship among the samples based on their expression values (Figure 2). A dendrogram was first constructed using the global expression dataset across the 16 samples with two major clusters, namely Cl-I and Cl-II. The dendrogram represented the clustering of resistant and susceptible genotypes together irrespective of treatment, hence further clustering was done only for eight infected samples to rule out the effect of genotype on treatments. This new clustering grouped six genotypes into three different clusters. To further validate the clustering obtained from a PCA using the expression dataset across the 8 samples, grouping was done based on PAC percentage and estimation of aflatoxin production which formed three groups for six resistant genotypes. Groups 1 and 2 consisted of a single genotype each, namely ICGV 91278 and ICGV 91284, respectively, while group 3 consisted of four genotypes, namely ICGV 91315, ICGV 91324, ICGV 93305, and ICGV 94379. J 11 was used as a resistant check and JL 24 was used as a susceptible check (Figure 2). Group 1 genotypes have PAC ≤ 2 μg/kg and AP ≤ 10 μg/kg, group 2 have PAC ≤ 2 μg/kg, AP ≤ 2 μg/kg and group 3 have PAC ≥ 40 μg/kg, AP ≤ 2 μg/kg. J 11 was used as a resistant check (PAC < 2 μg/kg; AP ≤ 0 μg/kg) and JL 24 was used as susceptible check (PAC > 90 μg/kg; AP > 940 μg/kg).

### 3.3. Differential Expressed Genes under Control and Infected Conditions

Group-wise comparisons were made using RNA-seq data between group 1: resistant J 11(I) vs. resistant genotypes (ICGV 91278, ICGV 91315, ICGV 93305, ICGV 91284, ICGV 94379, ICGV 91324) in infected condition, group 2: susceptible JL 24 vs. resistant genotypes (ICGV 91278, ICGV 91315, ICGV 93305, ICGV 91284, ICGV 94379, ICGV 91324) in infected condition, group 3: resistant J 11(C) vs. resistant genotypes (ICGV 91278, ICGV 91315, ICGV 93305, ICGV 91284, ICGV 94379, ICGV 91324) under control conditions, and group 4: susceptible JL 24 vs. resistant genotypes (ICGV 91278, ICGV 91315, ICGV 93305, ICGV 91284, ICGV 94379, ICGV 91324) under control conditions.

**Figure 3:**
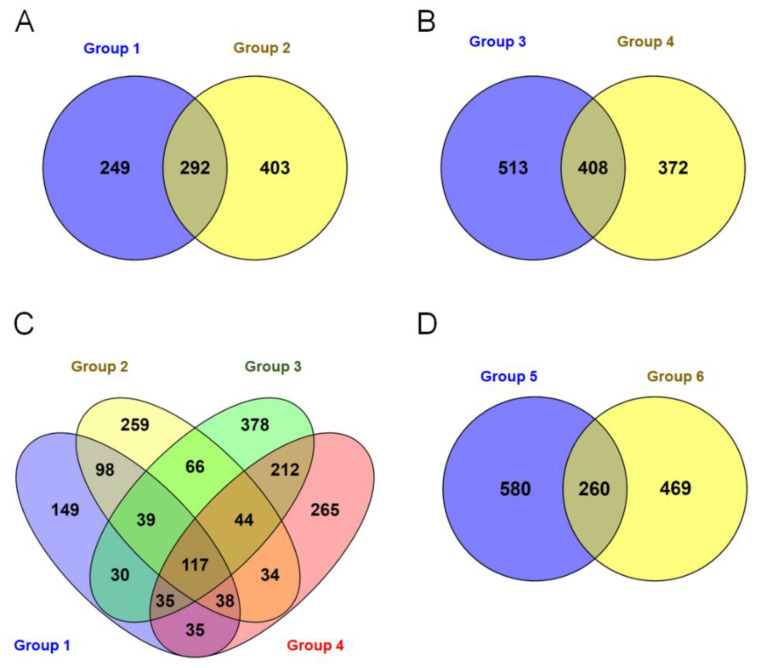
Differential expression of genes (DEGs) of groundnut genotype under control and infected conditions in response to *Aspergillus flavus*. (**A**) Group 1: moderately resistant J 11(I) vs. resistant (ICGV 91278, ICGV 91315, ICGV 93305, ICGV 91284, ICGV 94379, ICGV 91324) infected condition, Group 2: susceptible JL 24 vs. resistant (ICGV 91278, ICGV 91315, ICGV 93305, ICGV 91284, ICGV 94379, ICGV 91324) in infected condition, (**B**) Group 3: resistant J 11(C) vs. resistant (ICGV 91278, ICGV 91315, ICGV 93305, ICGV 91284, ICGV 94379, ICGV 91324) in control conditions, and group 4: susceptible JL 24 vs. resistant (ICGV 91278, ICGV 91315, ICGV 93305, ICGV 91284, ICGV 94379, ICGV 91324) under control conditions. (**C**) Number of differentially expressed transcripts between group 1, group 2, group 3 and group 4 and (**D**) group 5: susceptible JL 24 vs. (J 11, ICGV 91278, ICGV 91315, ICGV 93305, ICGV 91284, ICGV 94379, ICGV 91324 under control conditions, group 6: susceptible JL 24 vs. (J 11, ICGV 91278, ICGV 91315, ICGV 93305, ICGV 91284, ICGV 94379, ICGV 91324) in infected condition.

**Figure 4:**
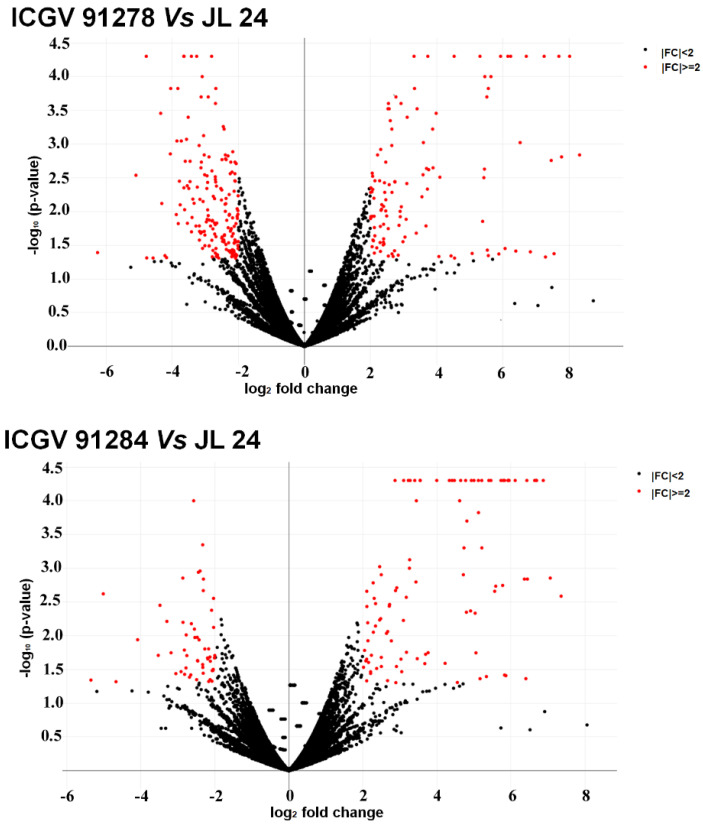
Volcano plot demonstrating the genes that were differentially expressed between susceptible (JL 24) and resistant genotypes (ICGV 91278, ICGV 91284) under infection conditions. Investigation and representation of DEGs were performed for developing Volcano plot. This plot is between log_2_ (fold change) and −log_10_ of the *p*-value on the x-axis and y-axis, respectively. Here, each dot is representing a gene and black and red coloured dots represent non-significant and significant genes, respectively.

A total of 541, 695, 921, and 780 differentially expressed transcripts were detected in group 1, group 2, group 3 and group 4, respectively (Figure 3A–C). Comparing these transcripts across the groups, 149 differentially expressed transcripts were specific to Group 1. Likewise, 259, 378 and 256 differentially expressed transcripts were detected specific to group 2, group 3 and group 4 respectively. Further, 117 unique differentially expressed transcripts were found common across the four groups (Figure 3C). Additionally, 840 and 729 differentially expressed transcripts were identified upon comparing 7 resistant genotypes with the susceptible check (JL 24) under control conditions (group 5) and in infected condition (group 6), respectively (Figure 3D). Biological processes such as metabolic and cellular processes, single-organism processes, biological regulation, and localization were identified based on the GO annotation of these expressed transcripts. A volcano plot depicted significant up- and down-regulation of DEGs between ICGV 91278 (I) vs. JL 24 (I) (total 292; up 115; down 177) and ICGV 91284 (I) vs. JL 24 (I) (total 156; up 103 down 53) samples (Figure 4). Altogether 448 DEGs including 218 significantly up-regulated and 230 down-regulated with log_2_ fold change values ≥2 or ≤−2, respectively, were identified (Figure 4) between susceptible check vs. infected genotypes.

### 3.4. Functional Annotations and GO Assignment

**Figure 5:**
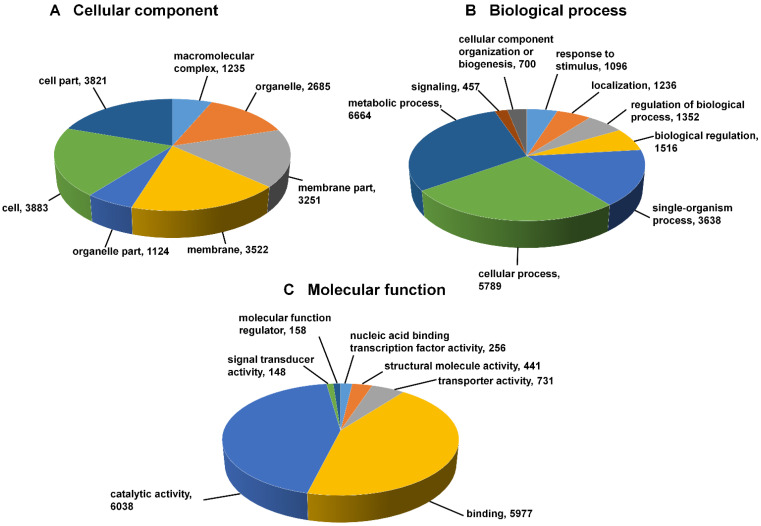
Distribution of gene ontology annotation assigned by Blast2 GO. This figure describes the three GO categories: cellular component (**A**), biological processes (**B**), and molecular functions (**C**).

The putative functional annotation and GO terms were assigned to 56,239 transcripts using the NCBI non-redundant (nr) Viridiplantae protein database. Three categories were distinguished: biological processes (72.83%), cellular processes (54.64%), and molecular functions (83.09%) (Figure 5; Table S2). In the cellular component category, cell, cell part, membrane and membrane part were the most important. Additionally, 7186 transcripts could be assigned and mapped to 138 different pathways in the KEGG information database (Table S3). The vast majority of the transcripts were mapped to the pathways related to purine metabolism (1063), thiamine metabolism (581), biosynthesis of antibiotics (546), and starch and sucrose metabolism (241). The complete list of pathways that were assigned to genes has been provided in the supplementary Table S3. This study identified 57 transcription factor (TF) families which include 6570 genes. The majority of these TFs were categorized into bHLH (719), MYB-related (479), NAC (437), and FAR1 family protein (320), among others.

### 3.5. Spatial Transcript Expression and Identification of Transcription under Control and Infected Conditions

**Figure 6:**
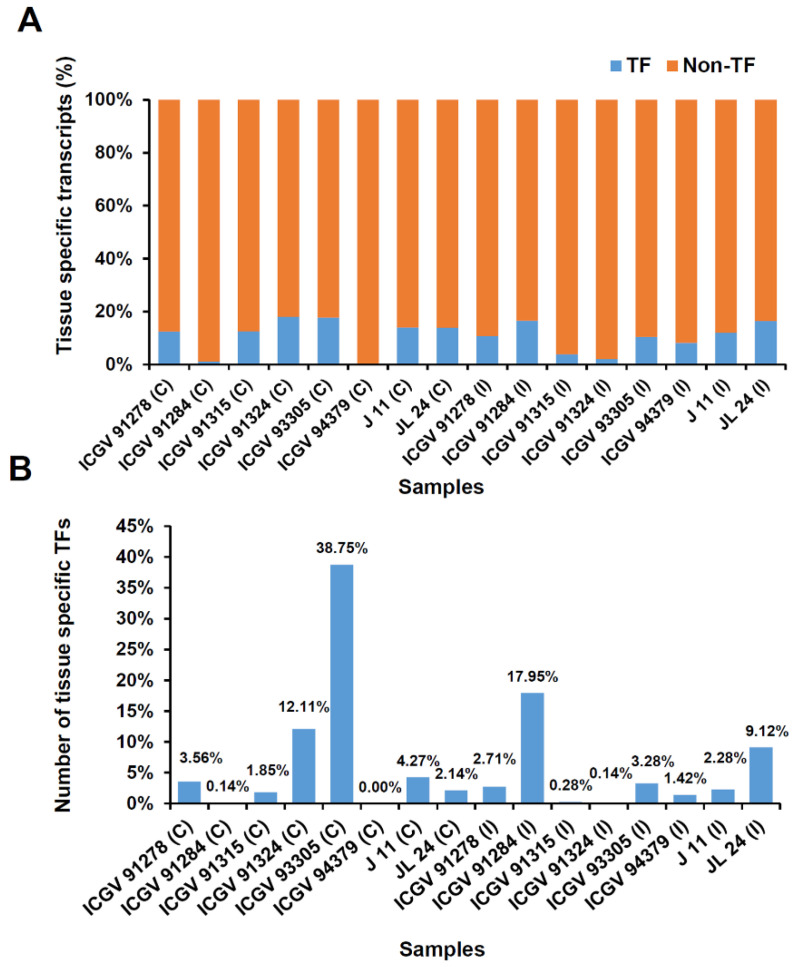
Spatial transcript expression identified in 16 samples. The figure shows the number of sample or genotype specific transcript in all 16 samples. Tissue specificity index (τ) was calculated to identify the genotype specific transcript. In the present study, transcripts with τ ≥ 0.9 were considered as sample specific. (**A**) Blue color represents sample specific transcript encoding transcription factors (TF) and orange color represents Non-TF transcripts. (**B**) The figure shows the distribution of sample specific transcript encoding transcription factors in all 16 samples.

A total of 4692 genes exhibited spatial transcript expression in 16 samples, including TF and non-TF encoding genes (Figure 6a). The largest numbers of sample specific transcripts were detected in ICGV 93305 (1533 genes), ICGV 91284 (762 genes), and ICGV 91324 (472 genes). Across samples, (38.75%), ICGV 91284 (17.95%) and ICGV 91324 (12.11%) had the highest number of the TF encoding genes (Figure 6b), mostly dominant TFs like bHLH family proteins (719), ERF family proteins, and NAC family proteins. In resistant genotypes, the expressed TFs belonging to basic helix–loop–helix (bHLH) DNA-binding superfamily protein were the biggest group followed by MYB (479), NAC (437), and ERF (309) family proteins. In susceptible samples, GATA family proteins, MYB related family proteins, bHLH family proteins, and bZIP family proteins were the most significant group of expressed TFs.

### 3.6. Pathways Genes Affecting during Host–Pathogen Interaction

**Figure 7:**
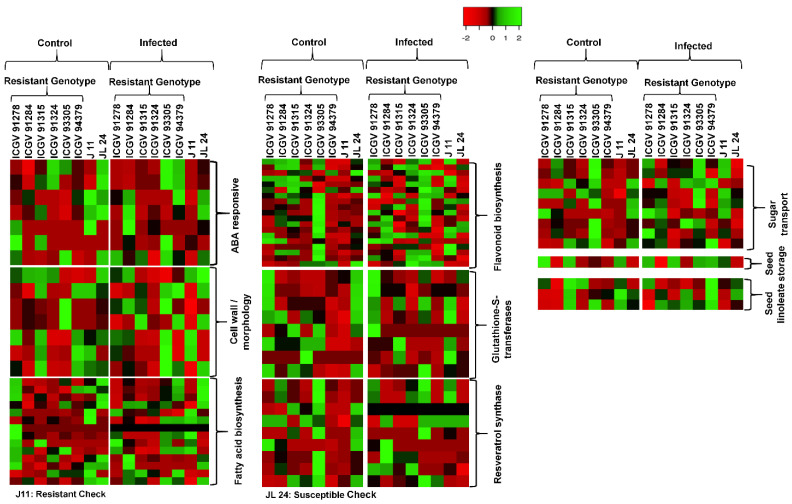
Heat map of different pathways of genes affected in 8 groundnut genotypes, both under control conditions and after infection with *A. flavus.* Some selected genes altered their expression in all of the pathways due to different pre-harvest aflatoxin contamination. Expression of genes involved in the glutathione-S-transferase, flavonoid biosynthesis, fatty acid biosynthesis, ABA responsive genes, resveratrol synthase, and seed linoleate gene expression is plotted. The color code corresponds to the FPKM value of the transcripts, increase from red to green. Genotype and treatment labels are shown at the top of the figure.

**Figure 8:**
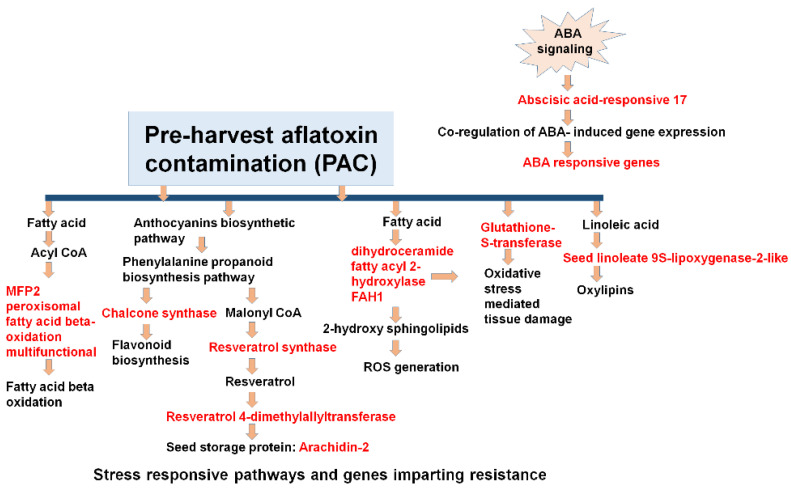
PAC is largely facilitated by moisture and heat stress during pod development in a green house. The study indicated an ABA-responsive *ABR17* gene belonging to the PR-10 class has been activated in plant–pathogen interactions during PAC. The components represented in red have an important role in host–pathogen interactions in this study, showing highly up-regulated patterns in resistant genotypes. Several pathways and TFs such as fatty acid biosynthesis, flavonoid biosynthesis, seed linoleate gene expression, resveratrol synthase, and glutathione-S-transferase play an important role in genetic resistance for PAC. The components in black represent the resistant pathways and other components which help in host-plant defense signaling.

The pathway examination was performed to examine the molecular function of the DEGs/genes expressed during the host–pathogen interactions (Table S3). During the analysis, 7186 DEGs were mapped to 137 different pathways for aflatoxin infection. These pathways represent three different types of metabolic pathway, i.e., primary, secondary, and other metabolic pathways. The primary metabolic pathways mainly involve lipids, amino acids, carbohydrates, and vitamins. During this study, the most heavily influenced carbohydrate metabolism pathways included glycolysis, glyconeogenesis, glycogen metabolism, citric acid, starch and sugar metabolism. Likewise, the major lipid metabolism pathways affected during PAC contamination included fatty acid biosynthesis and degradation, sphingolipid metabolism and glycerolipid metabolism. Similarly, the most affected amino acid metabolic pathways during *A. flavus* infection included tyrosine, tryptophan, biosynthesis of phenylalanine, glutathione and several other pathways related to the metabolism of vitamins. Among secondary metabolism pathways, the most affected included carotenoids, steroids, flavonoids, cutin, and wax in addition to shikimate derivative dependent pathways. This shikimate derivative dependent pathway includes biosynthesis phenylpropanoid, flavonoid stilbenoid, diarylheptanoid, isoflavonoid, and cyanoamino acid metabolism. During PAC contamination and aflatoxin production, some selected pathways and their genes/DEGs were affected under both control and infected conditions (Figure 7). These important pathways include fatty acid biosynthesis, flavonoids biosynthesis, seed storage, sugar transport, resveratrol, seed linoleate, cell wall-related genes, ABA-responsive genes and 9-LOX related genes (Figure 8 and Table 2) (Table S4).

## 4. Discussion

The molecular mechanism correlated with the PAC mechanism is not yet clearly well-defined and the availability of high-quality reference genomes provides a great opportunity for improving our current understanding. *A. flavus* infection and aflatoxin production are reported to be controlled by a combination of factors like reactive oxygen species (ROS), LOX genes, carbohydrate and fatty acid biosynthesis pathway factors, defense proteins like pathogenesis related etc. [47,48,49,50,51]. In this context, by the transcriptomic profiling of the eight different resistant and one susceptible groundnut genotypes under control and infected conditions, the current examination yields a conclusive understanding of the molecular mechanisms involved with PAC in the seed.

Previous studies indicate that defense responses related to PAC mechanism is a multi-layered cycle which incorporates transcriptional control, the induction of various resistant genes, cell wall component factors, generation of ROS species, and PR related proteins that prompt opposition against pathogen attack. Many TFs, especially MYB and MYB related, bHLH, WRKY, NAC, AP2 TFs play vital roles in developing a defense mechanism [52]. Further, systemic acquired resistance (SAR) plays an essential role during the initial pathogen attack [53] and is also responsible for the expression of pathogenesis-related proteins (PR-proteins) which directly act as a defense against various pests and pathogens [54]. Several pathways play an important role in providing defense reactions against pathogen attack and these pathways might be salicylic acid (SA), jasmonic acid (JA) and ethylene signaling [55]. These pathways play a vital role during signaling regulation.

The current investigation distinguished around 137 non-redundant pathways that were influenced by the 7186 DEGs, contributing to various pathways like pyruvate metabolism (169), fatty acid biosynthesis (61), TCA cycle (79), aflatoxin biosynthesis (21), glycolysis/gluconeogenesis (17), starch, and sucrose metabolism (241). Carbohydrate, fatty acid and amino acid metabolism are the primary metabolic pathways that are substantially affected. It was reported that glucose and carbohydrates are considered as an excellent substrate for both fungal growth and AF production [15]. Glucose is the supported carbon hotspot for *A. flavus*; therefore, it controls sucrose hydrolyzing enzymes that promote the *A. flavus* infection by providing a consistent stock of supplements to the pathogen [56]. The previous studies suggested that pathogens create a favorable environment for their development by manipulating the metabolism of crops [57]. In *A. flavus* infected maize, two core pathways such as starch degradation and hexose mobilization were reported [58].

Pathogenesis-related proteins (PR proteins) play significant roles in the plant’s defense system [59]. The defensins are commonly antimicrobial peptides that have been associated with plants, and guard against different microbial assaults by collaborating with membrane multi-layer lipids for their natural action [60]. After the induction of infection, PR genes are activated and subsequently produce PR proteins. In the infected seeds, the expression of the *pathogenesis-related 2-like* gene (ARD_1670) and *pathogenesis-related PR-4 isoform X2* (ARP_1078) showed up-regulation in the resistant genotypes (ICGV 91278, ICGV 94379, J 11) of groundnut seed, while the reverse was true under control conditions.

Similarly, for defensin genes in infected seeds, the expression of the defensin 1 protein (UN_2125) showed up-regulation in the resistant genotypes (ICGV 91278, ICGV 93305, ICGV 94379, J 11) of groundnut seed while the reverse was true under control conditions. Similarly, for defensin-2, under control conditions, the expression of the defensin 2 protein (UN_4369) showed up-regulation in the resistant genotypes (ICGV 91315, ICGV 93305, ICGV 94379) of groundnut seed while the reverse was true under normal conditions. A recent report also suggested significant reductions in aflatoxin production through a host-induced gene silencing (HIGS) approach by overexpressing antifungal defensins genes (*MsDef1* and *MtDef4.2*) (*aflM* and *aflP*) from the aflatoxin biosynthetic pathway in groundnut [61], indicating the importance of these genes in defense against *A. flavus* and the importance of deploying of genomic variation for crop improvement.

On the other hand, the flavonoids are phenylalanine-derived secondary metabolites that play a significant role in contributing to resistance against pathogens [62] and are also known to detoxify ROS produced by pathogens and the plant during infection [63]. Flavonoid biosynthesis pathways include the production of chalcone synthesized by *CHS*, *NAD(P)H-dependent 6-deoxychalcone synthase*, and chalcone isomerase. Here, we observed that the expression of *CHS* genes and chalcone-flavanone isomerase proteins during *A. flavus* infection was higher in the resistant genotype (J 11) as compared to JL 24 (susceptible genotype). In the infected seeds, the expression of chalcone isomerase (ARD_21521, ARD_21522, ARP_15581) and *NAD(P)H-dependent 6-deoxychalcone synthase* (ARD_13942, ARD_19144) showed up-regulation in the resistant genotype of groundnut seed while it the reverse was true under control conditions. The CHS protein was also found to be involved in salicylic acid signaling pathways during plant safeguard responses [64]. The expression of *CHS* is higher in resistant genotypes as compared to susceptible genotypes. Similar observations were made while studying the resistance mechanisms for in vitro seed colonization (IVSC) and aflatoxin production (AP) of *A. flavus* in the groundnut [9,65] which showed interconnection between different resistance mechanisms.

UDP-glucose flavonoid 3-O-glucosyltransferase 3 is a protein involved in the biosynthesis of secondary metabolites [66]. It plays an important role in anthocyanin biosynthesis and phenylpropanoid pathways. Probably, BOI-related E3 ubiquitin-ligase 3 plays a vital role in plant defense response mechanisms [67]. Under control conditions the expression of UDP-glucose flavonoid 3-O-glucosyltransferase 3 (ARD_12934, ARP_13077) showed down-regulation in the resistant genotypes (ICGV 94379, ICGV 91324, J 11) as compared to the susceptible genotype (JL 24) while in infected seeds, the expression of UDP-glucose flavonoid 3-O-glucosyltransferase 3 showed up-regulation in the resistant genotypes (ICGV 94379, ICGV 91324, J 11) of groundnut seed as compared to the susceptible genotype (JL 24).

Under control conditions, the expression of the *BRG3* (Probable BOI-related E3 ubiquitin- ligase 3) gene (ARD_2757, ARP 1913) showed down-regulation in the resistant genotypes (ICGV 91284, ICGV 93305, ICGV 94379, J 11) as compared to the susceptible genotype (JL 24) while in infected seeds, the expression of probable BOI-related E3 ubiquitin-ligase 3 showed up-regulation in the resistant genotypes (ICGV 91284, ICGV 93305, ICGV 94379, J 11) of groundnut seed as compared to the susceptible genotype (JL 24).

Aflatoxin is mainly produced by the utilization of fatty acid biosynthesis and sugar utilization through acetyl CoA and malonyl-CoA pathways through glucose catabolism [68]. Oxylipins, produced by oxygenase enzymes, regulate signaling pathways in fungi and also play a role during conidial growth in fungi [69]. In the infected seeds, the expression of oxylipin (ARD_10525 and ARP_3974) showed up-regulation in these resistant genotypes (ICGV 91278, ICGV 91324, and J 11) of groundnut as compared to JL 24, while it was the reverse under control conditions. These fatty acids also serve as key molecules that are engaged in plant defense in the development of cuticular elements and phytohormone jasmonic acids (JA). The immediate roles of fatty acids in plant defense have also been shown by modulating the response of basal, effector-triggered and systemic immunity [70]. Secondary metabolic pathways that include terpenoids, flavonoids, and steroid biosynthesis pathways have additionally been demonstrated to be influenced during the *Aspergillus* infection. Their role in plant defense is mostly related to their strong anti-oxidative properties.

The *ABR1* ethylene-responsive transcription factor is a significant factor because it is an ABA response repressor [71]. In the infected seeds, the expression of ABA-responsive *ABR17* (ARP_24754 and UN_4369) exhibited high-regulation patterns in resistant genotypes (ICGV 93305, ICGV 94379, and J 11) as compared to JL 24 (susceptible genotype), while under control conditions this gene showed down-regulation patterns in all the resistant genotypes of groundnut seeds. ABA interacts with *FUSCA3* which in turn regulates fatty acid biosynthesis and oil production [72,73]. The high expression in aflatoxin-contaminated seeds indicates that this gene is a candidate susceptibility factor for contamination before harvest. This finding is reinforced by the parallel observation that ABA signals repressed by this gene were also down-regulated in contaminated groundnut seeds. A previous study also highlighted the importance of the two pathways fatty acid biosynthesis and abscisic acid (ABA) in addition to the susceptibility factor ABR1 as a repressor of ABA signaling, in permitting PAC [39].

In summary, this study provided a deeper understanding of the molecular mechanism for pre-harvest aflatoxin contamination in groundnut in addition to the complex molecular interaction between groundnut and *A. flavus*. This study provided insights into the different genes and pathways that play key roles in inducing plant defense mechanisms for PAC resistance in groundnut. It has been clearly observed how several DEGs were activated or repressed by *A. flavus* infection in group 2 (ICGV 91284) and 3 (ICGV 91284, ICGV 91315, ICGV 93305, ICGV 94379); more DEGs were up-regulated in group 1 (ICGV 91278) than in group 2 (ICGV 91284) at every time point. More importantly, several genes/pathways were found highly up-regulated in PAC (pre-harvest aflatoxin contamination) mechanisms in infected seeds. It is important to note that several biotic and abiotic stresses such as insect and nematode damage, drought and heat stress, poor cultural and farming practices exacerbate PAC and affect pod yield and quality in the field. In response, several resistant genes were either up-regulated or down-regulated to combat the *A. flavus* infection in the field. This study also indicated the vital role of ABA-responsive *ABR17*, which co-regulates the genes of ABA responsive elements during drought stress, in providing resistance against *A. flavus* infection. It belongs to the PR-10 class and is also involved in several plant–pathogen interactions. Several other biotic and abiotic stress components activated several pathways and TFs such as fatty acid biosynthesis, flavonoid biosynthesis, seed lineolate gene expression, chalcone synthase, 9s-LOX, resveratrol synthase, and glutathione-S-transferase; defense related-genes which play important roles in genetic resistance to PAC. These genes were highly up-regulated in the resistant genotype controlling PAC resistance. These genes and pathways were working in a co-regulated manner to provide resistance against *A. flavus* infection, and can be used for enhancing PAC resistance through genomics-assisted breeding.

## Figures and Tables

**Table 1:** Summary of the sequence data generated from 16 RNA libraries through Illumina sequencing.

Sample Name	Total Raw Reads (Millions)	Total QC Reads (Millions) (Q > 20)	Overall Read Mapping (%)
ICGV 91278 (C)	65.12	64.11	94.90
ICGV 91284 (C)	62.03	58.89	94.80
ICGV 91315 (C)	65.42	63.76	94.80
ICGV 91324 (C)	60.15	58.79	94.40
ICGV 93305 (C)	55.24	53.00	94.20
ICGV 94379 (C)	82.69	80.57	94.30
J 11 (C)	44.60	42.62	94.80
JL 24 (C) *	57.92	56.55	94.20
ICGV 91278 (I)	58.17	56.52	94.20
ICGV 91284 (I)	60.47	58.71	93.90
ICGV 91315 (I)	76.38	73.46	94.30
ICGV 91324 (I)	58.63	56.95	94.40
ICGV 93305 (I)	61.50	59.63	94.40
ICGV 94379 (I)	55.80	54.31	94.10
J 11 (I)	54.94	52.38	94.40
JL 24 (I) *	52.58	51.67	89.90
Total	971.63	941.93	-
Minimum	44.60	42.62	89.90
Maximum	82.69	80.57	94.90
Average	60.73	58.87	94.13

C—Control condition; I—Infected condition; * Susceptible check.

**Table 2:** Highly differentially expressed putative candidate genes for PAC in groundnut.

Parents	Gene ID	Annotation	Expression Values (FPKM)	
			Control	Infection
ICGV 91278	UN_2125	DEF2_Defensin 2	10520.9	20643
	ARP_1078	Pathogenesis-related PR-4 isoform X2	44.09	102.03
	ARD_1670	Pathogenesis-related 2-like	16.54	84.08
ICGV 91284	ARD_2757	Probable BOI-related E3 ubiquitin-ligase 3	10.73	15.04
	ARP_15581	Chalcone–flavonone isomerase 1A	6.84	3.29
ICGV 91315	UN_4369	ABA-responsive ABR17	16049.7	19023.1
ICGV 91324	ARP_13077	UDP-glucose flavonoid	16.9	19.36
	ARD_12934	UDP-glucose flavonoid	19.36	24.95
ICGV 93305	ARD_2757	Probable BOI-related E3 ubiquitin-ligase 3	9.56	24.75
	ARP_1078	Pathogenesis-related PR-4 isoform X2	47.09	128.9
	ARP_1913	Probable BOI-related E3 ubiquitin- ligase 3	25.9	63.27
	ARP_24754	ABA-responsive ABR17	11051.9	37685.6
	UN_2125	DEF2_Defensin 2	3289.82	19092.1
	UN_4369	ABA-responsive ABR17	16909.2	49108.9
ICGV 94379	ARD_12934	UDP-glucose flavonoid	11.62	16.89
	ARP_13077	UDP-glucose flavonoid	8.54	15.55
	ARD_1670	Pathogenesis-related 2-like	5.58	10.62
	ARD_2757	Probable BOI-related E3 ubiquitin-ligase 3	6.68	9.3
	ARP_1913	Probable BOI-related E3 ubiquitin-ligase 3	59.65	84.48
	ARP_24754	ABA-responsive ABR17	11532.5	27514.8
	UN_2125	DEF2_Defensin 2	1995.02	6773.65
	UN_4369	ABA-responsive ABR17	16056.3	35167.3
	ARD_13942	NAD(P)H-dependent 6 -deoxychalcone synthase	1.38	3.63
J 11 (R)	ARD_12934	UDP-glucose flavonoid	16.73	25.32
	ARP_13077	UDP-glucose flavonoid	7.83	24.47
	ARD_1670	Pathogenesis-related 2-like	2.2	49.04
	ARD_2757	Probable BOI-related E3 ubiquitin-ligase 3	11.33	17.58
	ARP_1078	Pathogenesis-related PR-4 isoform X2	0.54	30.46
	ARP_1913	Probable BOI-related E3 ubiquitin- ligase 3	27.13	44.57
	ARP_24754	ABA-responsive ABR17	9734.37	12586.6
	UN_2125	DEF2_Defensin 2	1683.17	13333.2
	ARD_21521	Chalcone–flavonone isomerase 2	0	1
	ARD_21522	Chalcone–flavonone isomerase 1B-1	0	0.7
	ARP_15581	Chalcone–flavonone isomerase 1A	2.28	4.82
	ARD_13942	NAD(P)H-dependent 6 -deoxychalcone synthase	2.19	4.64

## Data Availability

All sequencing data generated have been deposited into National Center for Biotechnology Information (NCBI) Sequence Read Archive (SRA) database under the BioProject ID: PRJNA732524.

## Supplementary Materials

The following are available online at https://www.mdpi.com/article/10.3390/jof7060413/s1. Table S1: Expression values (log_2_ transformed FPKM) of genes expressed in 16 samples, Table S2: GO of biological process, molecular function and cellular function in genes expressed in 16 samples, Table S3: List of pathways identified in KEGG database, Table S4: Important pathways controlling PAC in groundnut.
